# ‘The true me’: Unravelling the dual narrative of borderline personality disorder and autistic spectrum disorder

**DOI:** 10.1111/bjc.70042

**Published:** 2026-03-08

**Authors:** Robin Iliffe‐Lewis, Alison M. Bacon

**Affiliations:** ^1^ School of Psychology University of Plymouth Devon UK

**Keywords:** autism, borderline personality disorder, BPD, diagnosis, emotionally unstable personality disorder, EUPD

## Abstract

**Objectives:**

There is growing recognition that some individuals who receive a diagnosis of borderline personality disorder (BPD) are later diagnosed with autism. However, existing literature on this topic remains limited. This study aimed to explore the experiences of individuals diagnosed with BPD prior to autism, how they made sense of these diagnoses, navigated clinical systems and learnt to manage the complex challenges associated with this diagnostic sequence.

**Methods:**

Thirteen (6 male, 6 female, 1 non‐binary) adult participants took part in semi‐structured interviews exploring their diagnostic journeys. Reflexive thematic analysis was used to develop a nuanced understanding of their experiences.

**Results:**

Three themes were constructed: (1) The Limitations and Challenges of Diagnostic Overshadowing, capturing how participants felt their BPD diagnosis failed to fully explain their difficulties, prompting them to seek further assessment; (2) Stereotyping and Misconceptions, exploring the impact of stigma and stereotyping, both internalized and encountered within clinical settings; and (3) Learning to Cope in a New World, describing how receiving an autism diagnosis enabled participants to reframe past experiences and develop new, often sensory‐informed coping strategies.

**Conclusion:**

These findings underscore the need for diagnostic processes that are open, curious and sensitive to overlapping presentations, and attentive to how historic stereotyping may shape clinical decision‐making. They also highlight the importance of post‐diagnostic support that empowers individuals to understand and adapt to their neurodivergence, fostering growth rather than perpetuating shame.

**Practitioner points**

**Implications for Clinical Practice**

- Adults with both BPD/EUPD and autism may interpret their diagnostic histories in ways that prioritize identity coherence and self‐understanding, highlighting the need for collaborative formulation.
- Clinicians should be cautious about attributing all emotional or interpersonal difficulties to BPD; autistic traits may be present but previously overshadowed.
- Autism‐informed adaptations (e.g. sensory considerations, structured communication) may increase engagement and therapeutic benefit, even within established treatments such as DBT.
- Recognizing the role of stigma in BPD diagnosis can support more validating clinical conversations and reduce diagnostic barriers.

**Cautions and Limitations**

- Participants often viewed autism as a more validating explanation of their experiences, but this does not preclude meaningful BPD–autism comorbidity; clinical assessment should consider both.
- The sample consisted of adults who actively sought reassessment, which may limit generalisability to individuals with different help‐seeking patterns.
- Diagnostic narratives were self‐reported; clinicians should triangulate such accounts with developmental history and multi‐informant evidence.
- Findings reflect UK‐based assessment pathways, which may differ in service accessibility and diagnostic criteria elsewhere.

## INTRODUCTION

The reported prevalence of autism spectrum disorder (ASD) has increased substantially over recent years, largely explained by changes in diagnostic and reporting practices (Hansen et al., 2015; Hodges et al., 2020). Historical understanding of autism, defined by rigid DSM‐III guidelines, limited recognition of individuals who did not fit traditional stereotypes (Howlin, 2021). However, with greater insight into autism, the diagnostic criteria has been updated to represent this new knowledge; criteria for social deficits were changed from ‘pervasive lack of responsiveness to other people’ to ‘persistent deficits in social communication’ (American Psychiatric Association [APA], 2013; Happé & Frith, 2020). Subsequently, the rate of adult autism diagnosis has increased (Happé et al., 2016) with a 787% increase in recorded diagnoses between 1998 and 2018 (Russell et al., 2022). This has led some researchers to speculate about a ‘lost generation’ of autistic adults, perhaps disguised by misdiagnosis (Lai & Baron‐Cohen, 2015).

There are substantial age‐related differences in the number of people diagnosed with autism, with children being more likely to be diagnosed than adults (O'Nions et al., 2023). O'Nions et al. (2023) suggest an estimated 55% of autistic people without intellectual disabilities in the United Kingdom are undiagnosed. The impact of undiagnosed autism can lead people to feel different, isolated or alien (Stagg & Belcher, 2019), which, in turn, can lead to higher rates of depression, anxiety and psychotic symptoms (Aggarwal & Angus, 2015). The complication of autism combined with mental health difficulties can hinder access to support, leaving people stuck at home and feeling like a burden to others (Camm‐Crosbie et al., 2019). Furthermore, stigma surrounding both mental health and autism means these people are doubly disadvantaged when seeking support (Crane et al., 2019; Nicolaidis et al., 2015).

Overlapping diagnostic symptomology can further exacerbate complexities surrounding autism diagnosis in adulthood, notably the symptomology of borderline personality disorder (BPD) (Dell'Osso et al., 2023; McQuaid et al., 2024). BPD was classified as emotionally unstable personality disorder (EUPD) in the ICD‐10 (World Health Organization, 1992) before being replaced by a dimensional classification of personality disorder in the ICD‐11 (World Health Organization, 2019). Despite these changes, BPD remains the term used within the DSM‐5 and continues to be widely adopted across much of the research literature and clinical practice (Jones, 2023). In this paper, the term ‘BPD’ is used for consistency with DSM‐5 and the predominant terminology in the evidence base, while acknowledging that EUPD is also a valid and still commonly used term. Although autism shares diagnostic features with various psychiatric conditions, BPD presents a distinctive diagnostic challenge due to core areas of overlap including emotional dysregulation, sensory sensitivities and challenges related to self‐identity and self‐perception (Au‐Yeung et al., 2019; Dudas et al., 2017). These overlaps can confuse clinicians which may result in ineffective treatment and worse outcomes for patients (McQuaid et al., 2024).

The complexities are illustrated in a study by Kentrou et al. (2021), who investigated 1019 participants' psychiatric conditions predating, co‐occurring and after their diagnosis of autism. Two hundred participants had received a personality disorder diagnosis (albeit not exclusively BPD) before being diagnosed with autism. After an autism diagnosis, 150 participants no longer met the criteria for personality disorder, with 85 participants having comorbid personality disorder and autism diagnoses. These findings suggest some traits interpreted as personality disorder may instead reflect underlying autistic characteristics. Autistic individuals, particularly those diagnosed in adulthood, are at increased risk of experiencing stigma, misunderstanding and interpersonal trauma, factors which may further contribute to behaviours and emotional difficulties commonly associated with BPD (Hull et al., 2020; McQuaid et al., 2024; Tint & Weiss, 2018).

Recent qualitative studies have begun to illuminate the experiences of individuals diagnosed with BPD prior to receiving an autism diagnosis and have highlighted significant challenges within diagnostic and therapeutic processes. Tamilson et al. (2024) interviewed autistic women who described their initial diagnosis as stigmatizing and disempowering, often imposed without their agreement or understanding. Clinicians often saw their distress as intentional or manipulative, attributions often linked to BPD (Aviram et al., 2006) leading to diagnostic overshadowing, thus resulting in exclusion from autism assessment pathways. As a result, participants were often offered treatments, such as dialectical behavioural therapy (DBT), an evidence‐based intervention for BPD which uses group work to support management of emotions and interpersonal relationships (Chapman, 2006). This encouraged masking behaviours, which participants found invalidating and, in some cases, harmful to their mental health (Kerns et al., 2016; Raymaker et al., 2020). By contrast, receiving an autism diagnosis was typically described as ‘life‐changing’, offering a sense of validation, self‐understanding and a shift from shame to acceptance. Similarly, in a further qualitative study Powell et al. (2024), participants discussed how their self‐harm (a coping strategy used to manage sensory overload and internal distress) was interpreted by clinicians as a symptom of BPD, even though self‐harm is frequently reported in women with autism (Rebbettes & Bacon, 2025; Tollerfield et al., 2023) and therefore cannot be considered a differentiating feature. This contributed to their misdiagnosis and years of ineffective or distressing treatment. The distinction between BPD and autism can also be blurred through the interplay between experiences of trauma and resulting attachment difficulties. Insecure attachment in BPD, particularly the unresolved, preoccupied and fearful subtypes, results in instable interpersonal relationships (Fonagy, 2000). While insecure attachment may also be found in autism, often the relationship difficulties observed are linked to difficulties in navigating social dynamics. Notably, in Powell et al. (2024), participants did not typically identify with key features of BPD, such as fear of abandonment or interpersonal dependency. Instead, they described feeling content alone and often overwhelmed by social interaction. The subsequent autism diagnosis was experienced as an accurate account of their lived experience. Nevertheless, it is important to note that while these experiences suggest the presence of undiagnosed autism in people with BPD, the opposite may also be the case and the two may also co‐occur (Dell'Osso et al., 2023).

The present study contributes to this growing literature exploring the experiences of individuals diagnosed with BPD prior to receiving an autism diagnosis. While previous studies have begun to examine this trajectory, they focused exclusively on participants identifying as female. The current study expands on this work by including individuals of multiple genders and exploring not only participants' diagnostic experiences, but also the factors that led them to seek a second opinion and any learning that occurred post‐diagnosis. It explores the longer‐term processes through which participants come to reinterpret their past, advocate for their needs and reframe their identities in light of an autism diagnosis.

## METHODS

### Participants

Thirteen participants were recruited through social media groups and support organizations for people with BPD and/or autism. Six participants identified as male, six as female and one as non‐binary. The average length of time between diagnosis of BPD and autism was 10.1 months (Range: less than one month–36 months). Recruitment criteria required participants to be over the age of 18 years old, commit to an interview about their experiences and report clinical diagnoses of BPD or EUPD as well as autism. Demographic Information is provided in Table 1.

**TABLE 1:** Participant demographic data.

Pseudonym	Gender	Age range	Year diagnosed with BPD	Year diagnosed with autism	Ethnicity	Other diagnoses
Ethan	Male	45–54	2021	2024	White British	CPTSD
Imani	Female	25–34	2021	2022	Black African	None reported
Liam	Male	35–44	2018	2019	White and Black African	None reported
Amina	Female	25–34	2018	2019	White and Black African	None reported
Nia	Female	25–34	2018	2019	White and Black African	None reported
Jasper	Male	25–34	2023	2024	White and Black Caribbean	None reported
Malik	Male	25–34	2020	2020	Black African	None reported
Sophie	Female	25–34	2019	2019	White British	None reported
Hannah	Female	25–34	2015	2015	White British	None reported
Alex	Non‐Binary	25–34	2015	2018	White British	CPTSD
Imogen	Female	25–34	2018	2018	White British	None reported
Andre	Male	18–24	2021	2023	White and Black African	None reported
Kwame	Male	25–34	2021	2021	Black African	None reported

### Procedure

Ethical approval was granted by the authors' University Research Ethics & Integrity Committee, reference number 2025–5439‐7693. On expressing interest, participants were provided with an information sheet showing details of the study and explaining their ethical rights, including the right to withdraw, voluntary participation, and that results of the study would be published in an academic journal. They completed an online consent form and returned it by email. The interview date was confirmed and then participants were sent a Zoom link or called at the agreed time. Semi‐structured interviews were conducted either online or by telephone according to participant preference. Participants were reminded of the purpose of the interview, their ethical rights and that they could skip any uncomfortable questions. Further verbal consent was obtained at this stage. Participants who interviewed online could turn their camera off if it felt more comfortable. The interviews were then recorded either via Zoom or call recording. The interview schedule was designed by the first author, reviewed by the second author and by a person with lived experience of both BPD and autism who was not involved in the study as a participant. Any amendments following this were agreed through discussion. The questions covered the participants' experiences of being diagnosed, their emotional response to these diagnoses, any stigma they had faced and any coping strategies they had learnt following both diagnoses. A full copy of the interview schedule is provided in the Appendix. At the conclusion of the scripted questions, participants were invited to offer any further reflections if they wished and were thanked for their time. Participants were sent an information sheet with reminders of ethical rights, details of how to withdraw their data if they wished, together with details of some online support services in case they would be useful. They also received a £10 Amazon Gift Card as a thank you. The audio recordings were transcribed using Microsoft Word, the transcripts were checked for accuracy and corrected where necessary. These transcripts were imported into NVivo 14 for data analysis. All participants were given pseudonyms to protect their confidentiality.

### Data analysis

Analysis followed the six‐phase approach to reflexive thematic analysis suggested by Braun and Clarke (2022). In the familiarization process, the first author read the data set multiple times before then coding the data by identifying relevant portions and developing code labels to represent the meanings. NVivo was used as a tool to organize and engage with the data. The second author reviewed the initial codes and offered feedback. The first author grouped similar codes to develop initial themes, which were then reviewed by the second author. An inductive approach to analysis was used, and themes were actively developed through reflexive engagement with the data to represent participants' experiences.

## REFLEXIVE POSITION/STATEMENT

The first author is a trainee clinical psychologist without a diagnosis of autism or BPD; however, they do have experience of working in mental health care, particularly with people diagnosed with both BPD and autism. They approached this analysis aware of their outsider position. Their positionality regarding diagnosis in clinical practice is nuanced. On one hand, they believe that diagnostic labels can provide individuals with a sense of explanation and validation for their experiences, which may be experienced as relieving or containing. On the other hand, diagnoses can contribute to diagnostic overshadowing, stigma and stereotyping. The author's clinical practice is primarily formulation‐driven, aiming to help individuals develop psychological understandings of their experiences rather than relying solely on diagnostic labels. Research supervision with the second author was used to refine and understand the first author's positionality when analysing data and identifying transcripts.

The second author is an academic with an interest in neurodiversity. She has a diagnosis of ADHD herself and recognizes how diagnosis can offer explanation and validation of both experience and identity.

## RESULTS

**FIGURE 1:**
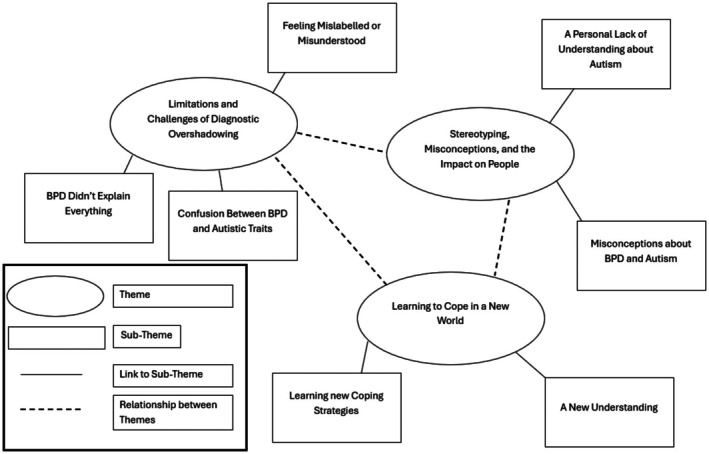
Thematic map.

Three themes were identified from the interviews. These describe the range of experiences participants had after receiving their diagnoses. Several participants felt their BPD diagnosis did not fully explain their difficulties, leading them to seek out further explanation. They faced misunderstanding and stereotyping from the public and professionals. Ultimately, receiving their autism diagnosis was viewed by most participants as positive, enabling them to reflect on their experiences in a positive way and learn new ways of coping. The relationships between the themes reported are illustrated in the thematic map (Figure 1).

### Theme 1: ‘I felt like something was missing’: The limitations and challenges of diagnostic overshadowing

Participants described how they felt their BPD diagnosis did not fully explain their difficulties and subsequently sought out an alternative explanation. The diagnosis of BPD led to unique challenges, as some professionals would focus on certain aspects of difficulty while ignoring others. This misunderstanding affected the care that some participants received.

#### Subtheme: BPD did not explain everything

Many participants described how they felt their BPD diagnosis did not fully explain their difficulties. A strong theme was constructed about the sense of feeling different and how this led to participants subsequently seeking out a further diagnosis of autism.

I had always felt different, but I didn't really realise I might be autistic until I learned more about autism in adults and particularly in people assigned female at birth. I struggled with, you know, sensory overload, social difficulties and interacting, but my emotional difficulties led professionals to diagnose the BPD first. It wasn't until I sourced a second opinion after feeling that BPD didn't explain all of my experiences that I was assessed for autism. I guess I needed to be sure of everything and what was really the problem, so I needed to, you know, get more opinions from people, from professionals. I just wanted some sort of clarity. (Amina)

Even after my BPD diagnosis, I felt like something was missing, like it didn't fully explain all of my struggles. I always felt different from others like I was on the outside looking in. Social interaction really drained me and I struggle with a lot of sensory overload and I had a deep need for routine and predictability, but no one ever suggested autism before. Probably because I had learnt to mask so well. I actually started researching autism in adults and people who were diagnosed late. The more I read the more I related, especially when it's related to things like difficulty understanding social norms or intense special interests. A lot of sensory sensitivities, actually, emotional struggles, regulation. In ways I felt different from just BPD I even had to sort an autism assessment, which was a longer frustrating process. Many professionals really still view autism to all the kids stereotypes, so I had to advocate for myself. (Malik)

I went through all of my teenage years and all of that before not knowing why I didn't fit in. Not knowing why all of the different types of therapy that I tried through the NHS didn't work. So I think I researched it in the beginning. It had been brought up to my parents growing up a few times, but it was never pursued by anyone and I've been in the mental health system since I was 14… 19 I think is when I looked into it and understood it for myself, and then saw parts of myself in other people's experiences when they have been diagnosed with autism. (Alex)

#### Subtheme: Confusion between BPD and autistic traits

Many traits of BPD (such as relationship difficulties or emotional instability) can end up mimicking or masking traits of autism, and vice versa.

One major challenge is that many health professionals don't understand, don't fully understand, *[the]* overlap between BPD and autism. Like BPD makes me crave connections, but autism makes socialising exhausting. (Amina)

Sometimes you might have similar symptoms such as emotional dysregulation, which makes it difficult to determine what drives the behaviours when it's that similar. (Jasper)

One participant went further in wondering whether their BPD diagnosis exists or if it is better explained by their autism diagnosis. ‘It's a difficult one. Sometimes I sit here and think maybe BPD doesn't exist. Maybe all along it was autism’. (Ethan).

#### Subtheme: Feeling mislabelled or misunderstood

Some participants spoke about feeling as though their BPD diagnosis led to difficulties in accessing treatment or that they were being pushed to inappropriate treatment.

In the beginning I was pushed to have DBT, which is very neurodivergent unfriendly and obviously that didn't work and then it made me feel like I wasn't trying hard enough. There was another time, a few years ago, when I was suffering really badly with an eating disorder and I was refused treatment… because of the BPD diagnosis. (Alex)

Having both diagnoses simultaneously made finding the right treatment very difficult. Because many mental health services focus on either autism or BPD, but not both together. I've had to advocate for myself a lot to get this right support. (Malik)

While participants often described their BPD diagnosis as failing to account for their lived experiences, attempts to seek reassessment were frequently shaped by broader societal and clinical misconceptions. The following theme explores how stereotyped views of both BPD and autism held by professionals created barriers to recognition and accessing support. Furthermore, some participants describe how despite their autism diagnosis, they felt confused or scared in part due to this stereotyping.

### Theme 2: Stereotyping, misconceptions and its impact on people

This theme continues with the sense of confusion and challenge that an ill‐fitting diagnosis can have. Historic stereotypes about BPD and autism are pervasive and led to ongoing misconceptions about how participants may act or feel. Furthermore, these stereotypes affect how some participants feel about their autism diagnosis.

Due to stereotyped views about autism and how it often presents, participants felt challenges from both the wider population and professionals.

#### Subtheme

Misconceptions about both BPD and autism were commonly identified.

Another misconception is that people with BPD are untreatable. Or for autism people think we are antisocial or lacking empathy, but that's not really the case. (Liam)

I don't really share much about my diagnosis with people, but a few of them will just be like, ‘people diagnosed with autism, they are not really intelligent, like intellectually, they are just disabled’ yeah, they are antisocial. (Imani)

People assume it [BPD] means they're manipulative, overly dramatic or impossible to have a relationship with… For autism, the biggest misconception is that autistic people lack empathy, which isn't true. I just process emotions differently. (Malik)

Participants described how prevailing misconceptions about what autism ‘should’ look like shaped others' perceptions, and in turn influenced their own experiences. These assumptions often led to doubt or dismissal from others, particularly when participants did not fit the stereotype. *‘When you think people are not going to believe that you're autistic. If some people don't look autistic’*. (Ethan). *‘It's mostly like, the older generation who have got like old school ideas of what autism looks like’* (Alex).

These misconceptions were not just interpersonal; participants also described how stigma and misinformation about BPD and autism could lead to reduced opportunities and further social exclusion.

After being diagnosed I tried to get another job so I can help myself. I was asked about my health, and I mentioned autism and then they said they cannot take me. I was discriminated because of my health condition. (Nia)

I couldn't mingle with people because I felt they were going to stigmatise me and isolate me… I was doing things on my own, I couldn't really interact with people. (Sophie)

These misconceptions sometimes led to challenges in participants receiving support for their mental health.

Well, unfortunately my experience with healthcare professionals has been mixed. Some focus too much on the BPD diagnosis and dismiss my autism related struggles… Others don't believe I have BPD because I don't fit this stereotypical image. (Amina)

I have had doctors dismiss my autism because I have a BPD diagnosis or only assume my struggles are due to BPD rather than recognising my sensory and processing difficulties. (Malik)

#### Subtheme: A personal lack of understanding about autism

While some participants described a sense of feeling their BPD diagnosis did not fully explain their difficulties. Others felt quite shocked after receiving their autism diagnosis in part due to confusion about autism and what it might mean for them. *‘*[After being diagnosed with autism] *It felt tricky, it wasn't easy to accept. I felt like it was something I had to fight… It took time for me to accept it’*. (Jasper)

But you know, going through, maybe social media or others kind of gathering information. People do say something about it. I did not really have a full knowledge about it. When I got diagnosed, that's why I know about autism… it is kind of difficult to come to accept it. (Andre)

I was confused. Like, I didn't know what autism means and how it would affect my life. At the moment I was sad… I was afraid that others may feel scared, and you know, uncertain about what the diagnosis means for my life and my future. (Hannah)

Despite the challenges created by stigma and misunderstanding, receiving an autism diagnosis offered many participants a renewed framework for making sense of their experiences. The final theme considers how participants' new understandings led to a greater sense of self‐compassion, and learning new strategies to manage their distress, particularly due to recognizing how important it is for them to focus on their sensory needs.

### Theme 3: The true me: Learning to cope in a new world

In this theme, participants described how their lives were changed following their diagnosis of autism. They learned new coping strategies, particularly sensory aids and increased predictability and routine. Having a diagnosis of autism enabled some participants to review some of their life experiences through a more positive, compassionate lens.

#### Subtheme: Learning new coping strategies

Participants described after being diagnosed with autism learning new coping strategies and a sense of gaining new insight into themselves.

I think my coping strategies have improved vastly over the last couple of months or so because I've learnt so much more about myself and had a lot more support. I've got like a weighted blanket, noise cancelling headphones or things like that which I'm finding really helpful. (Ethan)

All of these strategies, emotional regulation through sensory integration. I identify my social need and preference, I have a sensory integration plan to help regulate my emotion, a weighted blanket too. This really helps me to control my emotion. (Liam)

To manage both of them *[diagnoses]* is really everything. Everything in my life is structured. I have predicable routines that I use to actually help reduce my anxiety and emotional load. That is preparing before going into it, making a pattern like, how do I do my stuff. Also I use noise cancelling headphones too. I also avoid bright lights and wear comfortable clothing which help my sensory overload. I set boundaries in relationships to avoid emotional burnout. (Malik)

#### Subtheme: A new understanding

Participants felt after receiving their autism diagnosis that they gained new understanding, which in turn helped them to feel kinder towards themselves.

It explained why I struggled, and it helped me to learn how to navigate social situations. (Imogen).

I began to gain a little confidence in myself. After the diagnosis it was clearer. It helped with my social life and interactions. (Sophie)

This new understanding I had about myself helped me to be kinder to myself. Instead of blaming myself for struggling in social situations or feeling overwhelmed by change, I started recognising that my brain just worked differently. I began to focus my coping strategies to be more autism friendly rather than just managing BPD symptoms. (Malik)

It just helped me to come into the knowledge of truth about myself. It helped me develop a good habit and a compassion about myself that I didn't know could have existed. (Andre)

Knowing why I act, you know, the way I act helped me. It helped me to build self‐awareness. I knew my strengths and my weaknesses, my behavioural needs to manage it. (Hannah)

I realised that many of my struggles, meltdowns, difficulties with change, you know, black and white thinking, yeah, were autism rather than just emotional instability. It's allowed me to be kinder to myself, to reframe my experiences in a less judgmental way. (Amina)

Receiving an autism diagnosis provided participants with a new framework for understanding themselves in a more compassionate and coherent way. Many described the shift from self‐blame to self‐awareness as transformative, and this understanding enabled participants to implement new, sensory‐focussed coping strategies to help them reduce distress and improve emotional regulation.

## DISCUSSION

The three themes highlight the complex, often fraught experiences of receiving an autism diagnosis after a prior BPD diagnosis. A recurring thread across participants' narratives was the sense that the BPD diagnosis failed to fully capture their lived experiences, prompting many to seek further assessment. Their search was hindered by professional misunderstandings and diagnostic overshadowing, whereby pre‐existing labels led to autistic traits being overlooked or misattributed. The eventual autism diagnosis was experienced as a source of relief and clarity; it enabled a retrospective reframing of earlier difficulties, not as evidence of inherent dysfunction or something to be ashamed of, but as manifestations of neurodivergent needs. This shift in perspective fostered greater self‐compassion and a sense of legitimacy in their difficulties. Crucially, the insight gained post‐diagnosis empowered participants to develop and adopt more personalized coping strategies, particularly in relation to sensory processing and emotional regulation, developed from a more accurate understanding of themselves and their needs. While not directly reported above, participants also spoke about experiences such as diagnostic delay, the importance of casual and more formal support networks, and systemic barriers to assessment, these themes closely echoed findings previously reported in existing literature.

### Theme 1

The first theme explored participants' experiences of being diagnosed with BPD and the realization that this label failed to fully explain their difficulties. Many described feeling mischaracterised or misunderstood by their BPD diagnosis, particularly when traits that were later recognized as autistic, such as emotional intensity, sensory overwhelm or difficulties with social situations, had been previously interpreted as pathological or manipulative. While the symptom overlap between BPD and autistic traits has been acknowledged in previous research (Dell'Osso et al., 2023; McQuaid et al., 2024), participants reported that this was not well understood by clinicians, contributing to diagnostic overshadowing and, in some cases, the inappropriate withholding of treatment or further assessment. These findings align with Tamilson et al. (2024) and Powell et al. (2024) who similarly describe individuals being denied access to services on the basis of their BPD diagnosis. However, while Tamilson et al. (2024) and Powell et al. (2024) centre the experience of receiving an autism diagnosis following BPD, the present study further reports about the barriers participants faced in pursuing their autism diagnosis. Participants described having to seek second opinions from professionals, repeatedly asking for a referral, facing stereotyped views about autism and suggestions for assessment never being formally followed up. This highlights the challenges faced by individuals navigating a diagnostic system that may not only miss autism but actively dismisses its further consideration and assessment when a BPD label is present.

### Theme 2

The second theme explored participants' reflections on how limited or stereotyped views of both autism and BPD shaped their understanding of themselves and their diagnostic experiences. Participants described encountering misconceptions about autism that were often driven by outdated or narrow public ideas, for example, associations with intellectual disability, being antisocial or lacking empathy. While previous literature has highlighted how such stereotypes contribute to diagnostic delays, particularly in women (Bargiela et al., 2016; Hull et al., 2020), the present findings suggest that these assumptions may present diagnostic barriers across genders. In this study, participants of all genders described encountering and internalizing stigmatized ideas about what autism ‘looks like’. For some, this included a lack of awareness of the spectrum of autistic traits, which raised difficult emotions such as confusion, self‐doubt or sadness when autism was later diagnosed. These findings point to a broader societal misunderstanding that may inhibit self‐recognition and delay access to assessment for a wide range of individuals. By incorporating a gender‐diverse sample, this study highlights that misconceptions are pervasive, often becoming internalized by individuals and reinforced by others. These beliefs can undermine self‐belief, complicate the diagnostic journey and subsequently act as significant obstacles to receiving appropriate support.

Similarly, participants described experiencing stigma related to the BPD diagnosis, mirroring previous findings on clinician attitudes (Day et al., 2018; Klein et al., 2022). Several participants reflected on challenges with health care professionals who focused narrowly on their BPD diagnosis, at times dismissing or overlooking autism‐related difficulties. The growth of the neurodiversity movement in recent years is suggested to have shifted the perception of autism from a disorder to a difference and may therefore be reducing stigma (McVey et al., 2023). No such shift has occurred for BPD and clinician perceptions that individuals with BPD are untreatable, manipulative or incapable of sustaining relationships are still widely experienced (Navarre, 2025; Powell et al., 2024; Tamilson et al., 2024). The present study expands on previous findings by demonstrating how stigma and stereotyping around both autism and BPD intersect to shape diagnostic experiences.

### Theme 3

The third theme explored how participants made sense of themselves following their autism diagnosis. This new understanding prompted personal growth, greater self‐compassion and the development of more tailored coping strategies. For many, the diagnosis offered a valuable sense of insight into their longstanding difficulties. Rather than viewing themselves as difficult or dysfunctional, participants described beginning to understand how their brains work differently and doing so with increased self‐kindness. This shift in perspective helped them to further identify their sensory needs and implement new strategies to support emotional regulation and daily functioning, with an added justification from their new diagnosis.

While previous research has also documented the identity‐related benefits of receiving an autism diagnosis in adulthood, such as relief, validation and a sense of coherence (Leedham et al., 2020; Powell et al., 2024; Tamilson et al., 2024), the present study highlights how diagnosis can also offer a practical, embodied framework for managing distress. Participants described making meaningful changes to their day‐to‐day lives, including using noise‐cancelling headphones, weighted blankets, maintaining predictable routines and setting boundaries in relationships. For many, this shift enabled a more compassionate and effective approach to their own wellbeing, replacing earlier self‐criticism with a clearer understanding of how to live well within their sensory and emotional environment.

Collectively, these themes reflect the broad life experiences of participants, capturing both the challenges and sources of support they encountered throughout their diagnostic journeys. Many participants described a growing sense that their initial BPD diagnosis left questions unanswered, prompting them to seek further explanation. For some, an autism diagnosis followed relatively quickly; for others, the path was marked by significant barriers, such as professional gatekeeping. These obstacles were often rooted in historical misunderstandings and pervasive stereotypes surrounding both BPD and autism. Such misconceptions shaped not only how participants were perceived by others, but also how they viewed themselves. In some cases, this led to doubt or ambivalence about their subsequent autism diagnosis, accompanied by difficult emotions. Yet for others, the autism diagnosis offered a powerful sense of validation and a more compassionate lens through which to understand their experiences. This reframing enabled participants to learn about themselves in new ways, and to feel permitted, perhaps for the first time, to implement new strategies that supported their well‐being.

One notable thread in participants' accounts appears to be that of an autism diagnosis replacing that of BPD. While participants recognized the degree of possible symptom overlap and that this may have contributed to their initial BPD diagnosis, the overwhelming feeling seemed to be that this diagnosis was incorrect. The sense of relief and reclaimed identity on receiving a diagnosis of autism supports this. No participant suggested that they may actually have both conditions, and therefore, both diagnoses may be appropriate. Their comments suggest a desire to reject the possibility of having a personality disorder and to embrace autism as an alternative. Clinically, however, at the time of interview, participants had both diagnoses. In addition to the known stigma among health professionals (Navarre, 2025), research has also shown that public knowledge of such disorders is poor and subject to similar stigmas (Sheehan et al., 2016). While many participants clearly felt an autism diagnosis appropriate to explaining their experience, the pervasive aura of stigma around personality disorders may have contributed to their rejection of this diagnosis.

## CLINICAL IMPLICATIONS

The findings of this study propose several recommendations for clinical practice. The theme of diagnostic overshadowing highlights the importance of clinicians remaining open to reassessment, even when a prior diagnosis such as BPD has been made. While some participants found diagnostic labels helpful in validating their experiences, others felt these labels limited further exploration of their difficulties. Clinicians should be cautious of relying solely on existing diagnoses and instead adopt a comprehensive, person‐centred approach, drawing on the full range of assessment tools and clinical curiosity to ensure that relevant neurodevelopmental conditions, such as autism, are not overlooked. Complicating the diagnostic process were entrenched, outdated and stereotyped views of both BPD and autism. While shifts in broader public understanding may be gradual, promoting greater clinical awareness is essential. This includes recognizing the diversity of autistic presentations and challenging stigma associated with BPD. Participants in this study described how such assumptions not only contributed to delays in diagnosis but also compounded feelings of shame and self‐doubt, with a detrimental impact on mental health.

For clinical psychologists and psychotherapists, these findings highlight the central role of formulation as a collaborative and evolving process. Moving beyond a diagnostic framework alone, formulation can help clinicians and clients make sense of how features of BPD and autism interact, shaping emotional regulation, interpersonal experiences and self‐identity. Integrating contextual factors, such as trauma history, sensory sensitivities and social communication needs into a shared formulation, may help guide intervention planning and prevent the overapplication of disorder‐specific models that do not fully capture the individual's lived experience.

In this context, trauma history (or the absence of trauma) is of interest in exploring difficulties observed in both autism and BPD, which may blur diagnostic boundaries but have very different underlying explanations. People with autism are at increased risk of exposure to adverse life events (Hoover, 2015; Kildahl et al., 2019; van Lobregt‐ Buuren et al., 2021), and these are often linked to depression and anxiety (Griffiths et al., 2019). They report higher levels of adverse childhood experiences than those without autism (Berg et al., 2016), and as adults remain vulnerable to experiences such as bullying and interpersonal violence (Griffiths et al., 2019; Trundle et al., 2022). Furthermore, sensory sensitivity, social confusion, misunderstanding and rejection by others can lead to elevated anxiety, affecting resilience to cope with stressors (Wood & Gadow, 2010). Adults with ASD are also more than four times likely to be diagnosed with posttraumatic stress disorder (PTSD) than adults without ASD (Griffiths et al., 2019), further complicating clinical presentation. BPD is also associated with trauma history, with the sequelae often presenting similarly to symptoms of PTSD (Golier et al., 2003). Behaviours associated with insecure attachment, particularly the unresolved, preoccupied and fearful subtypes, may represent understandable and reasonable adaptive behaviours in response to interpersonal adversity. As such, conditions such as BPD can be considered disorders of social communication, rather than inherent personal traits. This suggests that BPD is not stable and intractable to treatment but can change, and difficulties be overcome when they are no longer perceived as helpful, such as in a therapeutic intervention which engenders epistemic trust (Luyten et al., 2021).

Furthermore, while DBT is widely recommended for individuals diagnosed with BPD, several participants in this study and existing literature suggest that standard DBT protocols may not always meet the needs of autistic people. This is partly due to the treatment's reliance on group‐based skills training, which can pose additional challenges for those experiencing social communication and interaction differences. The use of metaphorical or abstract language, a core feature of DBT, may also limit accessibility for individuals who process information more concretely. In addition, the sensory and cognitive demands of intense sessions can be overwhelming, particularly when coupled with an overstimulating group environment. These barriers may lead to disengagement or non‐attendance, which can be misinterpreted as ‘treatment resistance’, thereby worsening outcomes for the individual and perpetuating harmful stereotypes. Clinicians are therefore encouraged to consider adapting DBT as appropriate, such as offering one‐to‐one delivery, adjusting language or pacing sessions differently, to ensure interventions are inclusive and responsive to the needs of the individual. Such modifications can be effective, as evidenced in autism‐adapted CBT (Spain et al., 2015), though Cooper et al. (2018) have highlighted the importance of appropriate training for therapists in adapting evidence‐based treatment for autism. Most recently, evidence has emerged suggesting that DBT can also be successfully adapted to meet the needs of autistic clients (Keenan et al., 2024).

Finally, the third theme underscores the value of thoughtful post‐diagnostic support. For many participants, learning about their sensory needs and developing appropriate coping strategies was central to their growth following diagnosis. Formation of a positive autistic identity is often driven by external validation such as that perceived on diagnosis. Ongoing support is fundamental to the development of such an identity and the associated improvements in mental health and well‐being (Davies et al., 2024). Clinicians are encouraged to consider how services can facilitate this process, ranging from psychoeducation about autism and sensory regulation to the development of targeted, specialist support pathways. Recognizing diagnosis as not only a clinical event but also a process of personal adaptation may improve individual outcomes and help foster greater self‐understanding.

## LIMITATIONS

First, participants were self‐selecting and self‐reported their diagnoses. Verification of formal diagnoses would have been challenging due to restrictions in accessing medical records or participants missing the required documentation. Therefore, it was a deliberate methodological decision to prioritize lived experience to try and ensure voices were not excluded. However, this may mean the participant sample reflects a subset of individuals particularly motivated to share their stories, rather than the wider population. It may also exclude those who are less engaged with technology and the internet, or who do not seek support through online groups. Secondly, although recruitment materials were shared within UK‐based social media groups and UK‐based mental health support services, participants were not asked to confirm their location, meaning the study cannot draw conclusions about the influence of specific health care settings. This may, in part, account for the varied time intervals between diagnoses. Thirdly, although reflexivity was embedded throughout the analytic process, qualitative research is inherently shaped by the researcher's positionality. The findings should therefore be understood as a situated interpretation of the data, shaped by specific relational and epistemological contexts.

## FUTURE RESEARCH

Future research could explore the experiences of individuals who receive BPD and autism diagnoses in greater demographic depth. While this study included participants of multiple genders and a higher proportion of Black participants than typically seen in similar research, it did not explicitly examine how factors such as race, gender or cultural background may shape diagnostic experiences. Further qualitative work focused on these intersections may offer deeper insight into how systemic biases and structural inequalities influence diagnostic pathways and outcomes.

Additionally, future studies could explore the perspectives of clinicians involved in diagnostic assessment, particularly around the challenges of identifying autism in individuals with co‐occurring mental health difficulties. Understanding how professionals make diagnostic decisions, and how diagnostic overshadowing may operate in practice, could help improve training, reduce gatekeeping and support more nuanced assessment processes.

Finally, research could further investigate the role of post‐diagnostic support in helping individuals navigate and adapt to a new understanding of their identity. In particular, the development of sensory coping strategies and the impact of psychoeducation merit further attention, as they were described by participants as key to their personal growth and wellbeing following diagnosis.

## CONCLUSION

This study offers insight into the experiences of individuals diagnosed with BPD prior to receiving an autism diagnosis. Through reflexive thematic analysis, it explored how participants navigated complex diagnostic journeys, the impact of stigma and stereotyping from both the public and health care professionals, and how learning about their autism led to personal growth and the development of new coping strategies to mitigate distress. While participant experiences varied, strong patterns were identified around the perception that BPD did not fully explain their difficulties, the reframing of past experiences in light of an autism diagnosis and the sense of validation in adopting new strategies grounded in this understanding. These findings emphasize the need for diagnostic processes that are open, respectful and curious, ensuring that individuals' needs are heard, rather than overshadowed or dismissed. They also highlight the importance of post‐diagnostic support that informs and empowers individuals to integrate and act on their new self‐understanding. This study forms part of a growing area of research, one that should be respected and capitalized on, especially during a period of service reformation.

## AUTHOR CONTRIBUTIONS

**Robin Iliffe‐Lewis:** Conceptualization; investigation; writing – original draft; methodology; project administration; writing – review and editing; formal analysis. **Alison M. Bacon:** Conceptualization; writing – original draft; writing – review and editing; methodology; supervision; validation.

## FUNDING INFORMATION

No funding was received for this research.

## CONFLICT OF INTEREST STATEMENT

The authors declare no conflicts of interest.

## Data Availability

Research data are not shared.

## Appendix

1. Can you describe your initial reactions and emotions when you received your diagnosis of BPD/EUPD?
2. How did you experience your daily life, relationships and self‐perception after the BPD/EUPD diagnosis?
3. What led to your diagnosis of autism (ASC)? Can you describe the journey to this diagnosis?
4. How did you feel when you received your ASC diagnosis? How did it compare to your initial BPD/EUPD diagnosis in terms of emotions and understanding?
5. In what ways, if any, did the ASC diagnosis change your self‐identity or your perception of your experiences with BPD/EUPD?
6. Can you discuss any challenges or positive aspects of having both diagnoses?
7. How have these diagnoses influenced your interactions with health care professionals, family, friends or the broader community?
8. What strategies or coping mechanisms have you developed or learned to manage your BPD/EUPD and autism diagnoses?
9. Have you encountered any misconceptions or stigmas related to these diagnoses, and how have you responded to them?
10. In what ways do you feel these diagnoses have affected your access to treatment, support or services?
